# Response to Robin Bailey’s Letter to the Editor, ‘*Misclassifying and differentiating metacognitive therapy: conceptual and methodological issues in Stenzel et al.’*

**DOI:** 10.1017/S0033291725103012

**Published:** 2026-01-13

**Authors:** Kilian Leander Stenzel, Joshua Keller, Lukas Kirchner, Winfried Rief, Max Berg

**Affiliations:** 1 Department of Psychology, Division of Clinical Psychology and Psychotherapy, Marburg University, Marburg, Germany; 2 Clinical Psychology and Psychotherapy, Justus-Liebig-University Giessen, Giessen, Germany

Dear Editor-in-Chief,

We appreciate Dr. Bailey’s interest in our meta-analysis (Stenzel et al., 2025) and the opportunity to clarify his raised points (Bailey, 2025). We agree with some of his concerns, particularly that a potential misclassification of Metacognitive Therapy (MCT) should be discussed. Therefore, we present analyses addressing this point demonstrating that the impact on a central conclusion of our review – specialized treatments for repetitive negative thinking (RNT) have larger effects than general treatments – is negligible.

First, we acknowledge that MCT can be considered to target RNT. The Cognitive Attentional Syndrome, which MCT aims to modify, entails worry and rumination as components (Capobianco & Nordahl, 2023). In our original formulation of the introduction and the discussion, we relied on a summary concluding that ‘in the absence of prospective longitudinal studies or experimental manipulations of these variables, there is no evidence confirming the causal nature of meta-cognitive beliefs’ (Ehring & Watkins, 2008, p. 198). However, we agree that recent studies suggest that meta-cognitions possibly play a causal role for RNT (Capobianco, Heal, Bright, & Wells, 2019), which challenges our classification of MCT.

Second, concerns about the level of detail of one point of our preregistration procedure were raised. Our intention was to classify treatments as *RNT-specific* based on explicit statements in the methods sections of whether and how the treatment aimed to target RNT. However, we disagree with the conclusion that our decisions are a ‘material deviation from preregistered plans’ (Bailey, 2025, p.1). Preregistering this procedure (e.g. by listing all *RNT-specific* interventions *ad hoc*) is not trivial, if not impossible, as there may be randomized controlled trials (RCTs) applying treatments outside of their original theoretical framework. Therefore, we relied on openly available data (i.e. explicit textual statements) to classify studies. Hence, we disagree with criticisms regarding replicability, as this can hardly be resolved given the current – oftentimes insufficient – reporting practices concerning the intended influence of treatment kernels on mechanisms (Rief et al., 2024). Further, we still doubt whether ‘future reviews [can validly base] subgroup classifications on theoretically grounded mechanisms of change rather than surface textual criteria’ (Bailey, 2025, p. 2). This would leave classifications even more dependent on authors’ subjective judgments, and evidence suggests that postulated theoretical mechanisms of interventions account for little, if any, of the observed treatment effects of psychological therapies (Mulder, Murray, & Rucklidge, 2017). While these points could be further discussed, we believe to settle doubts about the interpretive robustness of our conclusion regarding ‘treatment specificity’ in the following.

We appreciate that Dr. Bailey pointed out that two MCT studies should be classified differently (Nordahl et al., 2018; Wells & Colbear, 2012). To evaluate the effects of this point, we reexamined the studies and reclassified them as *RNT-specific.* Two authors (K.L.S., M.B.) further revisited all previous decisions independent of each other according to our text-based classification and resolved inconsistencies by discussion. This yielded a substantial inter-rater reliability (*κ* = 0.61) and led to the consistent inclusion of Applied Relaxation and Emotion Regulation Therapy as *RNT-specific* treatments. For the reanalysis, 17 out of 67 treatment arms were reclassified. An updated version of the data table and analytic code is provided in the OSF repository (DOI: 10.17605/OSF.IO/WDBFJ). We then recalculated the subgroup difference between *RNT-specific* and *general* treatments based on the new classification. In the original analysis, *general* treatments had a medium effect size (*g = −*0.56), whereas *RNT-specific* treatments had a large effect size (*g = −*0.99) and the subgroup difference was significant, *Q*(1) = 10.04, *p* = .002. According to the updated classification, the medium effect size of the ‘general’ treatments became slightly smaller (*g = −*0.48). The *RNT-specific* treatments remained at a large effect size (*g = −*0.87). Likewise, the subgroup difference remained significant, *Q*(1) = 8.05, *p* = .005. Thus, the interpretation that ‘RNT-specific interventions seemed to outperform general approaches significantly’ (Stenzel et al., 2025, p. 9) remains unchanged. The difference appears robust and other central conclusions of the meta-study are not affected. In conclusion, we acknowledge that *MCT is an effective and RNT-specific treatment.*

In sum, we value the critique of potentially misclassifying some studies with regard to treatment specificity. However, after these studies were reclassified, the results of this subgroup comparison did not change notably. This is why one of our central conclusions, i.e. treatments targeting RNT specifically are more effective than general approaches (Stenzel et al., 2025), still holds. Being aware of the risk that incorrect meta-analytic interpretations pose to clinical decision-making, we hope that our corrections demonstrate not only the credibility of one of our central claims but also our openness to deal with critique in a constructive way.

Yours Sincerely

Kilian L. Stenzel, Joshua Keller, Lukas Kirchner, Winfried Rief, and Max Berg
